# Prevalence of Corneal Astigmatism and Anterior Segmental Biometry Characteristics Before Surgery in Chinese Congenital Cataract Patients

**DOI:** 10.1038/srep22092

**Published:** 2016-02-25

**Authors:** Duoru Lin, Jingjing Chen, Zhenzhen Liu, Xiaohang Wu, Erping Long, Lixia Luo, Zhuoling Lin, Xiaoyan Li, Li Zhang, Hui Chen, Jinchao Liu, Weirong Chen, Haotian Lin, Yizhi Liu

**Affiliations:** 1 State Key Laboratory of Ophthalmology, Zhongshan Ophthalmic Center, Sun Yat-sen University, Guangzhou, Guangdong, 510060, People’s Republic of China

## Abstract

The prevalence and the distribution characteristics of corneal astigmatism (CA) and anterior segment biometry before surgery in Chinese congenital cataract (CC) patients are not completely understood. This study involved 400 CC patients from the Zhongshan Ophthalmic Center enrolled from February 2011 to August 2015. Data on CA, keratometry, central corneal thickness (CCT) and anterior chamber depth (ACD) were measured by the Pentacam Scheimpflug System. The mean age of patients was 54.27 months, and the ratio of boys to girls was 1.53:1. The mean CA was 2.03 diopters (D), and 39.25% of subjects had CA values ≥2 D. The most frequent (71.8%) diagnosis was with-the-rule astigmatism. Oblique astigmatism was present in 16.2% of cases, and 12% of cases had against-the-rule astigmatism. The mean keratometry measurement of cataractous eyes in bilateral patients was significantly larger than that in unilateral patients. Girls had a larger mean keratometry but a thinner CCT than did boys. The CA, CCT, and ACD of cataractous eyes were significantly larger than those of non-cataractous eyes in unilateral patients. The CA, mean keratometry, CCT, and ACD in CC patients varied with age, gender, and laterality. Fully understanding these characteristics may help inform guidelines and treatment decisions in CC patients.

Congenital cataract (CC) is a primary cause of childhood blindness, which has become treatable in the past decade^1^,^2^. It is also one of the primary diseases affecting eye development, with the underlying mechanism involving defocus^3^,^4^,^5^,^6^ and form deprivation^7^,^8^. Several publications, including our previous studies, have reported that the development of axial length in pediatric patients with cataractous eyes is significantly different from that in healthy children^9^. However, reports on the prevalence and distribution characteristics of corneal astigmatism (CA) and anterior segment biometry in CC patients remain rare. Research on the developmental status of anterior segment biometry will help us fully understand the effect of CC on eye development beyond axial length, which will greatly improve the guidelines for CC treatment. Furthermore, keratometry and anterior chamber depth (ACD) are the most important indices in the accurate calculation of the required intraocular lens (IOL) power before surgery, whereas the variability in central corneal thickness (CCT) may affect the intraocular pressure (IOP) measurement in CC patients during the management of pediatric glaucoma^10^. The present study was designed to explore the prevalence of CA and the distribution of keratometry, CCT and ACD before surgery in Chinese CC patients and to compare data from the unilateral cataractous eye with those from the corresponding non-cataractous eye. Data on the developmental status of CA and anterior segment biometry in CC patients are of clinical significance to the guidelines for CC treatment, IOL power calculation, and IOP measurement.

## Results

Four hundred of the 476 (84.03%) CC patients with qualified measurements in both eyes were included. Of these eligible patients, 34% (136/400) presented with a unilateral cataract, and the remaining 66% (264/400) had bilateral involvements. The mean age was 54.27 ± 42.75 months, and the ratio of boys to girls was 1.53:1 (242:158).

**Figure 1:**
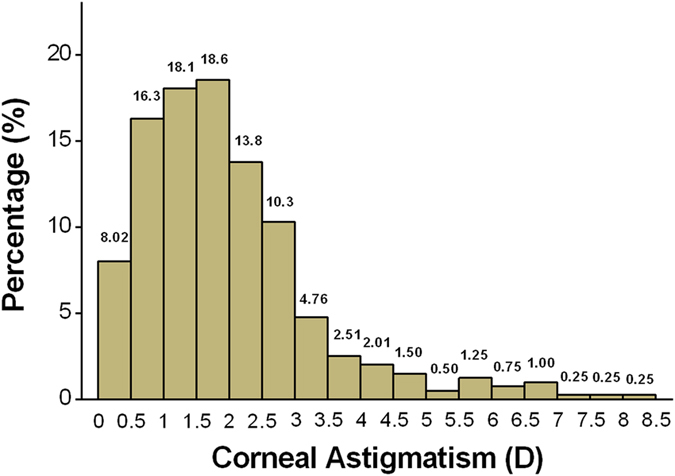
Distribution of corneal astigmatism in CC patients. Corneal astigmatism in CC patients largely (78.5%, 314/400) fell between 0.5 and 3 D. CC: congenital cataract; D: diopters.

**Figure 2:**
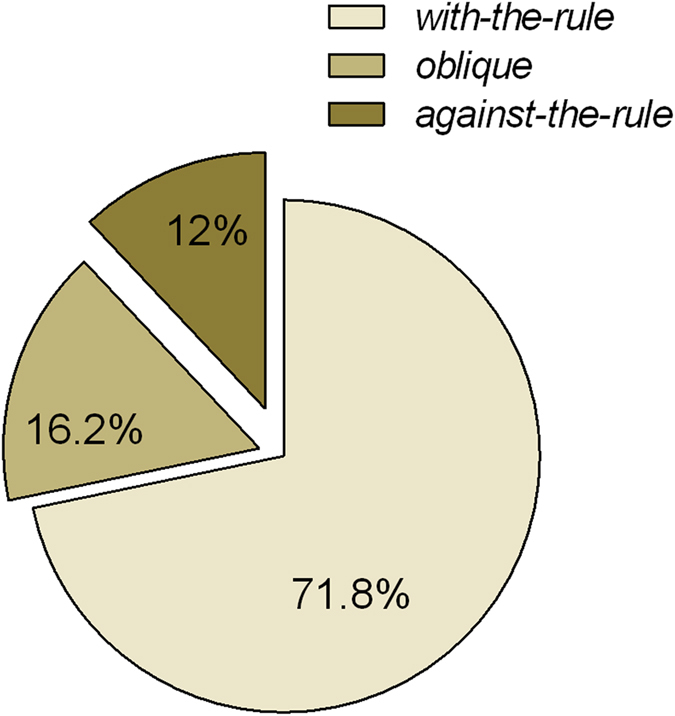
Constitution of the type of corneal astigmatism in CC patients. With-the-rule corneal astigmatism predominated among pediatric CC patients. CC: congenital cataract.

The CA values of CC patients largely (78.5%, 314/400) fell between 0.5 and 3 diopters (D), and the overall distribution of CA is shown in Fig. 1. The mean value of CA was 2.03 ± 1.46 D; 79% (316/400) of subjects had values of ≥1 D, and 39.25% (157/400) had values of ≥2 D. The most frequent (71.8%) diagnosis was with-the-rule astigmatism (WTR astigmatism) (steepest meridian at 180 ± 30 degrees). Oblique astigmatism (steepest meridian between 120 and 150 degrees or between 30 and 60 degrees) was present in 16.2% of cases, and 12% of cases had against-the-rule astigmatism (ATR astigmatism) (steepest meridian at 90 ± 30 degrees) (Fig. 2). Further description of the CA in patients by age group, gender and laterality is provided in Table 1.

Tables 2, 3, 4 show the distribution of mean keratometry, CCT, and ACD, respectively, by age group, gender and cataract laterality. The mean keratometry measurements of the cataractous eye in bilateral patients were significantly larger than those in unilateral involvements. Girls had a larger mean keratometry but a thinner CCT than did boys. No significant differences in ACD were found in CC patients by gender or laterality.

**Figure 3:**
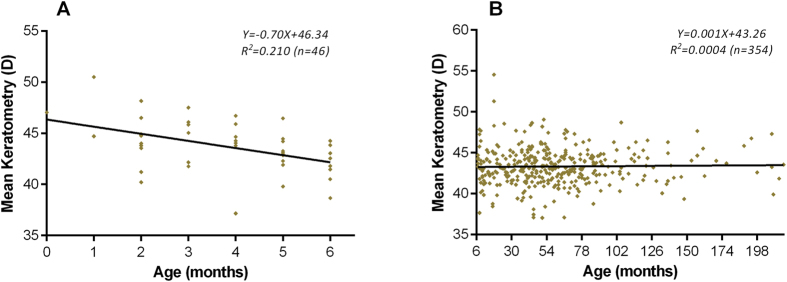
Scatterplots of age versus the mean keratometry value of all eyes. Panel (**A**) The trend line shows a linear decline in mean keratometry during the first 6 months of life. Panel (**B**) No significant change was found in the keratometry value with increasing age beyond 6 months. D: diopters.

**Figure 4:**
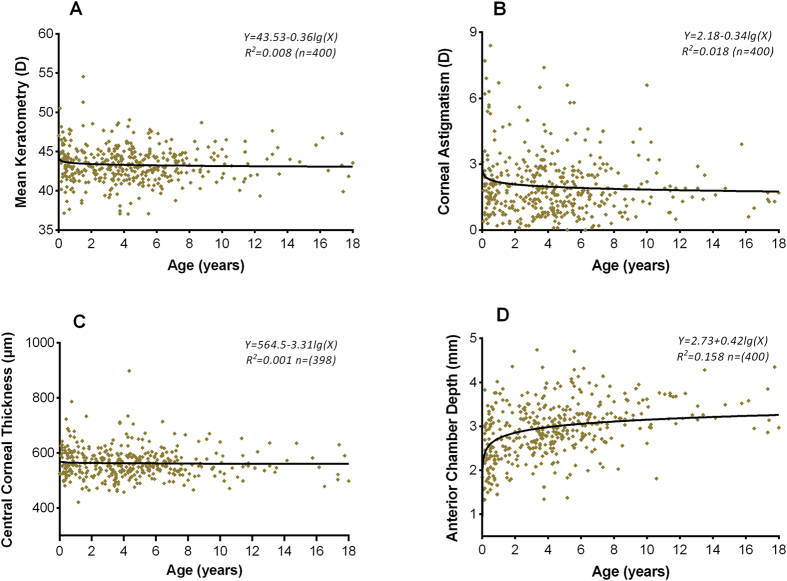
Scatterplots of mean keratometry, CA, CCT, and ACD in relation to age. Panel (**A**) (mean keratometry versus age), Panel (**B)** (CA versus age) and Panel (**C**) (CCT versus age) show little relationship between the respective variables and age. Panel (**D)** illustrates a logarithmic relationship between ACD and age. D: diopters; CA: anterior corneal astigmatism; CCT: central corneal thickness; ACD: anterior chamber depth.

Figure 3 shows scatterplots of mean keratometry versus age. A linear decline in mean keratometry with age was revealed in patients less than 6 months old, but this tendency was absent in older patients. Scatterplots of mean keratometry, CA, CCT, and ACD in relation to age are shown in Fig. 4. Panels A (mean keratometry versus age), B (CA versus age) and C (CCT versus age) show little relationship between their respective variables and age. Panel D illustrates a logarithmic relationship between ACD and age.

Table 5 shows the differences in mean keratometry, CA, CCT, and ACD between the unilateral cataractous eye and the clear counterpart eye. Larger CA, CCT, and ACD values were found for the affected eye.

The multiple linear regression equation of CA and mean keratometry, CCT, and ACD was shown as follow:


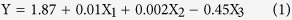


Notes: R^2^(adjusted) = 0.036, F = 5.97, P = 0.001 (P(X_1_) = 0.75, P(X_2_) = 0.18, P(X_3_) = 0.00); Y: CA, X_1_: mean keratometry, X_2_: CCT, X_3_: ACD.

## Discussion

Knowledge of the prevalence of preoperative CA and the associated characteristics of anterior segmental biometry can greatly enhance the guidelines for CC treatment and prognosis. However, few studies have used a large cohort of CC patients, perhaps due to the low incidence of CC, a lack of awareness of the importance of such data, the challenge of the necessary examination, and a lack of proper equipment. The Pentacam Scheimpflug System used in the present study can provide a 3-dimensional representation of the anterior segment of the eye, including comprehensive parameters, by rotating 180 degrees around the eye and capturing 25 single-slit images in less than 2 seconds^11^. Because of the merits of noncontact, rapid imaging and high quality, the success rate of examination in this study was relatively high (84.03%). In the present cohort, the mean CA in CC patients was larger than 2 D, and most patients had with-the-rule astigmatism. Girls had a larger mean keratometry but a thinner CCT than did boys, and a positive, linear relationship was found between ACD and age. Furthermore, the CA, CCT, and ACD values of the unilateral cataractous eye were significantly larger than those of the corresponding non-cataractous eye. This study is the first report on the prevalence of CA and the characteristics of the anterior segmental biometry in Chinese CC patients.

Previous studies have found a high rate of postoperative astigmatism in CC patients, although a number of cases reduced spontaneously over time^12^,^13^. Research on preoperative CA can be of great benefit in informing the guidelines for astigmatism treatment in CC patients. A previous study^14^ with a small sample size (62 patients) reported the distribution of CA in CC patients using an autorefract-keratometer. They found that the prevalence of CA ≥2 D in Japanese patients was 65.7%, and the most frequent diagnosis was WTR astigmatism. However, they did not describe the specific relationships between CA and patient demographics. In the present study using a large cohort of CC patients, similar findings regarding the prevalence and type of CA were revealed. Furthermore, we found that the prevalence of CA was unassociated with age, gender, and laterality. These findings demonstrate a markedly higher prevalence of CA (≥1.00 D, 79% in this study, and 89.9% in the study of Japanese patients mentioned above) in pediatric CC patients than in children without cataracts from Taiwan (13.3%)^15^, Northern Ireland (25–29%)^16^, and Australia (26.6%)^17^. Furthermore, the proportion of high astigmatism (≥2 D) in CC patients was higher than that reported for Tohono O’odham children, a native American tribe that has been documented with a high prevalence of astigmatism among preschool- and school-age members^18^. We also found that the affected eye of unilateral cataract patients had a higher CA than did the unaffected eye in 5 age groups. A higher CA in the eye with CC likely resulted from the abnormal development of the eyeball^19^. For those CC patients with high preoperative CA, the selection of an appropriate surgical procedure^20^, appropriate delay of the surgery^12^, the use of a small vitrectomy system^21^, and the adjustment of the type or location of the incision according to the CA reading^13^,^20^ may contribute to a decrease in the prevalence of postoperative astigmatism. In addition, a less favorable outcome with postoperative amblyopia treatment has also been reported in children with ATR astigmatism than those with WTR astigmatism;^22^ thus, a detailed explanation of the potential for poor postoperative visual function is needed.

In addition to information on astigmatism, knowledge of the distribution of other anterior segment variations, such as keratometry, CCT and ACD, can improve CC treatment and should be measured before surgery. Previous studies have described the distribution of keratometry in White and African American^23^, Nepalese^24^, Serbian^25^, and Italian children^26^. Trivedi and colleagues^23^ investigated keratometry in 299 pediatric eyes with cataracts in America and found that the average value was 45.39 D, with steeper keratometry in girls than in boys. They also found that the corneal curvature of the eye with the cataract was steeper in unilateral cases than in bilateral involvements and that the eye with the cataract had a significantly steeper cornea than did the corresponding eye in monocular cataract patients. However, until now, keratometry in Chinese children with cataracts has been unclear because keratometry varies with ethnicity^27^. In this study with a large Chinese cohort, we similarly found steeper keratometry in girls than in boys. However, the mean keratometry reading (43.37 D) was smaller than that found in other studies. This result may reflect the specific study population involved and the low proportion of younger children (only 11.5% of children were ≤6 months) in the present study, as infants were found to have a steeper corneal curvature. Similar to a previous finding, we found that newborns have a steeper corneal curvature than do older children; this curvature decreased with age and stabilized after 6 months of age^28^. This growth pattern of keratometry with age may reflect the compensatory changes in axial length that are required to maintain a constant state of refractive power^23^. Unfortunately, the anterior segment imaging and analysis system (the Pentacam in this study) does not provide the measurement of axial length. In addition, we found that the mean keratometry measurements of eyes in bilateral CC patients (6–18 years old) were significantly larger than those of the cataractous eye in unilateral involvements and that the keratometry value of the cataractous eye was the same as that of the healthy eye in unilateral patients, the latter of which contradicts previous findings^23^. In addition to age composition, different ethnicities and measurement techniques (keratometer vs. Pentacam) could account for these discrepancies among studies.

It is necessary to consider the CCT distribution in the management of pediatric glaucoma due to the effect of CCT on IOP measurement^10^. A small sample size of cataract patients (n = 30) and the multi-racial (≥5 races) composition of a previous study^29^ limited the scope of investigation of the relationships between CCT and the demographics of CC patients. The results of the present study of a large cohort of CC patients (n = 400) within a single racial group (Chinese) provide an overview of the distribution of CCT in Chinese pediatric CC patients. We found that the CCT of boys was thicker than that of girls, and a thicker CCT was revealed in the cataractous eye of 135 unilateral CC patients when compared with the contralateral healthy eye. Muir *et al.*^29^ found a trend of a thicker mean CCT in the cataractous eye than in the eye without a cataract (564 ± 34 μm vs. 552 ± 38 μm), although the difference became smaller after excluding eyes with an abnormal cornea from the cataract group. The CCT is usually thickest immediately after birth and then thins until approximately 3 years old^10^. Thus, we hypothesize that the thicker CCT in the eyes with a cataract may have resulted from the delayed development and maturation of the cornea.

ACD is a component of the axial length and is an indicator of the development of the anterior segment of the eyeball. In this study, the ACD of the cataractous eye showed a logarithmic relationship with age. This finding confirms that the increase in ACD with age may be a result of the increase in axial length due to the close relationship between ACD and axial length^30^. We also found that the ACD of the affected eye in unilateral cataract patients was larger than that of the fellow eye. However, this difference was limited to patients aged 2–6 years. Furthermore, both Twelker *et al.*^27^ and Trivedi *et al.*^30^ noted that girls had a shallower anterior chamber than did boys in both the healthy eye and the cataractous eye. Interestingly, in Chinese CC patients, we also found a smaller ACD in girls than in boys aged 7 months to 2 years, but no differences were observed in the other age groups. It is known that ACD varies with ethnicity^27^, but the relationship between ACD and gender in different races and whether boys have a deeper anterior chamber at all ages remain unknown. In addition, results of the multiple linear regression (eq. 1) showed that patients with short ACD were more likely to suffer larger CA than those with deeper anterior chamber. Therefore, more attention about the CA should be paid to those patients with shallow anterior chamber relatively easily found by a slit lamp examination.

The results of our study should be interpreted with caution. First, we only focused on the development of the anterior segmental biometry in eyes of Chinese CC patients and did not include measurement of axial length, which is another important indicator of eye development. Axial length will be included in our next study to provide a more comprehensive assessment of eye development. Second, a lack of fixation while measuring the anterior segmental parameters under chloral hydrate sedation in some patients may have introduced some error in our biometry readings^31^. However, massaging the eyeball to the primary position and referring to the quality index of the Pentacam image helped us to obtain reliable measurements in the absence of fixation. Despite these limitations, the results of this study describe the prevalence of CA and the overall distribution of anterior segmental biometry before surgery in a large cohort of Chinese CC patients. In conclusion, the CA of cataractous eyes in CC patients was significantly larger than that of eyes with a clear lens, and the most frequent type of astigmatism was WTR astigmatism. The mean keratometry, CA, CCT, and ACD in CC patients varied with age, gender, and laterality. The anterior segment biometry data of Chinese pediatric patients provided in the present study are of clinical significance to the guidelines for CC treatment, IOL power calculation, and IOP measurement.

## Patients and Methods

### Subjects

From February 2011 to August 2015, patients ≤18 years old with CC were recruited and enrolled in this study from the Zhongshan Ophthalmic Center (ZOC), one of the largest eye facilities in China and located in Guangzhou city in Southern China. Participants were eligible if the child was diagnosed with CC before surgery and lacked other ocular abnormalities, such as corneal diseases, lens luxation, glaucoma, retinal diseases, nystagmus, and nanophthalmos. The analyzed cataractous eyes were composed of the affected eye of unilateral patients and a randomly selected eye of bilateral patients. This study was approved by the Human Research Ethics Committee of the ZOC, Sun Yat-sen University. All procedures adhered to the tenets of the Declaration of Helsinki, and written informed consent was obtained from at least one parent of each patient.

### Anterior segment examination and parameters

Anterior segment parameters, including CA, mean keratometry, CCT and ACD, of CC patients were measured under an undilated pupil before surgery. These measurements were obtained using a 3-dimensional anterior segment imaging and analysis system (Pentacam HR, Oculus Inc., Wetzlar, Germany), which is a commercially available camera based on the Scheimpflug principle^11^. It provides a 3-dimensional representation of the anterior segment of the eye after rotating around the eye from 0 to 180 degrees and capturing 25 single-slit images in less than 2 seconds. All patients were tested by one experienced examiner (ZLL), and those patients who were unable to actively cooperate were sedated with 10% chloral hydrate (0.8 ml/kg, oral or rectal administration)^32^. The mean of 3 measurements that met the quality standards was calculated for each parameter.

CA, mean keratometry, CCT and ACD were the main parameters measured from the anterior segment of the eye and were defined in this study as follows. The difference between the steep keratometry and flap keratometry of the front surface of the cornea was defined as the CA, and the mean value of the steep keratometry and flap keratometry was defined as the mean keratometry. Furthermore, CCT was measured from the central anterior corneal epithelium to the central posterior corneal endothelium. The ACD, which is equivalent to the “ACD-lens” described in our previous publication^33^, was defined as the distance from the corneal endothelium to the lens epithelium.

### Statistical Analysis

Statistical analysis was performed using the Statistical Package for the Social Sciences (SPSS ver. 19.0, Chicago, IL, USA). Absolute frequency (n) and relative frequency (%) were used for the analysis of qualitative variables, and mean and standard deviation (mean ± SD) were used for the analysis of quantitative variables. The Kolmogorov-Smirnov test was used to evaluate the normality of the distribution for all variables. The t-test for independent samples was used to analyze the variables of cataract eyes, and the paired t-test was used to evaluate the differences between the affected eye and the fellow eye in unilateral patients. The relationships between the biometry data and age were analyzed based on Pearson correlation coefficients and regression analysis. The multiple linear regression was used to analyze the relationships between CA and mean keratometry, CCT, and ACD. A P-value < 0.05 was considered statistically significant.

## Additional Information

**How to cite this article**: Lin, D. *et al.* Prevalence of Corneal Astigmatism and Anterior Segmental Biometry Characteristics Before Surgery in Chinese Congenital Cataract Patients. *Sci. Rep.* **6**, 22092; doi: 10.1038/srep22092 (2016).

## Figures and Tables

**Table 1:** Corneal astigmatism measurements in the cataractous eye of pediatric patients by age, gender and laterality of the cataract.

	≤6 M	7 M to 2 Y	2–6 Y	6–18 Y	0–18 Y
Gender					
Girls	2.51 ± 1.83	2.02 ± 2.19	1.87 ± 1.21	1.78 ± 0.99	1.92 ± 1.41
	(n = 13)	(n = 25)	(n = 79)	(n = 41)	(n = 158)
Boys	2.71 ± 2.19	2.17 ± 1.39	1.97 ± 1.38	1.96 ± 1.22	2.10 ± 1.49
	(n = 33)	(n = 38)	(n = 107)	(n = 64)	(n = 242)
P	0.77	0.74	0.61	0.43	0.24
Laterality					
Bilateral	2.38 ± 2.04	1.81 ± 1.04	1.98 ± 1.38	2.00 ± 1.26	2.02 ± 1.42
	(n = 36)	(n = 41)	(n = 118)	(n = 69)	(n = 264)
Unilateral	3.62 ± 2.00	2.67 ± 2.52	1.82 ± 1.17	1.65 ± 0.80	2.05 ± 1.55
	(n = 10)	(n = 22)	(n = 68)	(n = 36)	(n = 136)
P	0.10	0.14	0.45	0.09	0.82
Total	2.65 ± 2.07	2.11 ± 1.74	1.93 ± 1.31	1.89 ± 1.13	2.03 ± 1.46
	(n = 46)	(n = 63)	(n = 186)	(n = 105)	(n = 400)

The data are presented as the means ± standard deviation (SD). Bold data are significant at P < 0.05. M: months; Y: years.

**Table 2:** Mean keratometry measurements in the cataractous eye of pediatric patients by age, gender and laterality of the cataract.

	≤6 M	7 M to 2 Y	2–6 Y	6–18 Y	0–18 Y
Gender					
Girls	44.13 ± 2.58	44.66 ± 3.24	43.87 ± 2.04	43.75 ± 2.03	43.99 ± 2.31
	(n = 13)	(n = 25)	(n = 79)	(n = 41)	(n = 158)
Boys	43.54 ± 2.50	42.71 ± 2.18	42.78 ± 2.22	43.12 ± 1.88	42.96 ± 2.17
	(n = 33)	(n = 38)	(n = 107)	(n = 64)	(n = 242)
P	0.48	**0.01**	**0.001**	0.11	**0.00**
Laterality					
Bilateral	43.95 ± 2.43	43.37 ± 2.35	43.44 ± 2.30	43.65 ± 1.98	43.55 ± 2.24
	(n = 36)	(n = 41)	(n = 118)	(n = 69)	(n = 264)
Unilateral	42.84 ± 2.72	43.69 ± 3.54	42.91 ± 2.01	42.83 ± 1.82	43.01 ± 2.32
	(n = 10)	(n = 22)	(n = 68)	(n = 36)	(n = 136)
P	0.22	0.66	0.11	**0.04**	**0.02**
Total	43.71 ± 2.51	43.48 ± 2.80	43.24 ± 2.21	43.37 ± 1.95	43.37 ± 2.28
	(n = 46)	(n = 63)	(n = 186)	(n = 105)	(n = 400)

The data are presented as the means  ±  standard deviation (SD). Bold data are significant at P < 0.05. M: months; Y: years.

**Table 3:** Central corneal thickness measurements in the cataractous eye of pediatric patients by age, gender and laterality of the cataract.

	≤6 M	7 M to 2 Y	2–6 Y	6–18 Y	0–18 Y
Gender					
Girls	538.92 ± 35.59	570.44 ± 77.90	545.46 ± 41.38	548.29 ± 40.83	549.61 ± 48.86
	(n = 13)	(n = 25)	(n = 79)	(n = 41)	(n = 158)
Boys	576.33 ± 53.68	571.34 ± 52.83	573.15 ± 59.11	567.44 ± 48.87	571.78 ± 54.56
	(n = 33)	(n = 38)	(n = 105)	(n = 64)	(n = 240)
P	**0.03**	0.96	**0.00**	**0.04**	**0.00**
Laterality					
Bilateral	570.17 ± 52.71	570.39 ± 68.49	559.09 ± 54.40	557.22 ± 41.56	561.87 ± 53.63
	(n = 36)	(n = 41)	(n = 117)	(n = 69)	(n = 263)
Unilateral	549.90 ± 47.15	572.09 ± 54.01	565.06 ± 53.20	565.22 ± 55.37	565.13 ± 53.17
	(n = 10)	(n = 22)	(n = 67)	(n = 36)	(n = 135)
P	0.28	0.92	0.47	0.41	0.57
Total	565.76 ± 51.74	570.98 ± 2.68	561.26 ± 53.90	559.96 ± 46.65	562.98 ± 53.43
	(n = 46)	(n = 63)	(n = 184)	(n = 105)	(n = 398)*

The data are presented as the means  ±  standard deviation (SD). Bold data are significant at P < 0.05. M: months; Y: years. *Central corneal thickness data were not available for 2/400 eyes.

**Table 4:** Anterior chamber depth measurements in the cataractous eye of pediatric patients by age, gender and laterality of the cataract.

	≤6 M	7 M to 2 Y	2–6 Y	6–18 Y	0–18 Y
Gender					
Girls	2.24 ± 0.55	2.45 ± 0.51	2.92 ± 0.54	3.24 ± 0.52	2.87 ± 0.61
	(n = 13)	(n = 25)	(n = 79)	(n = 41)	(n = 158)
Boys	2.48 ± 0.63	2.83 ± 0.72	2.99 ± 0.58	3.19 ± 0.48	2.95 ± 0.62
	(n = 33)	(n = 38)	(n = 107)	(n = 64)	(n = 242)
P	0.23	**0.02**	0.42	0.60	0.25
Laterality					
Bilateral	2.34 ± 0.63	2.68 ± 0.69	2.90 ± 0.52	3.24 ± 0.45	2.88 ± 0.61
	(n = 36)	(n = 41)	(n = 118)	(n = 69)	(n = 264)
Unilateral	2.65 ± 0.51	2.67 ± 0.64	3.06 ± 0.62	3.15 ± 0.58	2.99 ± 0.63
	(n = 10)	(n = 22)	(n = 68)	(n = 36)	(n = 136)
P	0.17	0.98	0.07	0.41	0.08
Total	2.41 ± 0.61	2.68 ± 0.67	2.96 ± 0.56	3.21 ± 0.50	2.92 ± 0.62
	(n = 46)	(n = 63)	(n = 186)	(n = 105)	(n = 400)

The data are presented as the means ± standard deviation (SD). Bold data are significant at P < 0.05. M: months; Y: years.

**Table 5:** Comparison of the anterior segment variations of the affected eye and fellow eye in unilateral cataract patients.

	Age	n	Affected Eye	Fellow Eye	P
Km (D)	≤6 M	10	42.84 ± 2.72	42.32 ± 1.79	0.639
	6 M to 2 Y	22	43.69 ± 3.54	42.40 ± 2.22	0.075
	2–6 Y	68	42.91 ± 2.01	42.93 ± 1.65	0.901
	6–18 Y	36	42.83 ± 1.82	43.12 ± 1.44	0.144
	0–18 Y	136	43.01 ± 2.32	42.85 ± 1.72	0.392
Astig (D)	≤6 M	10	3.62 ± 2.00	1.88 ± 1.27	**0.028**
	6 M to 2 Y	22	2.67 ± 2.52	1.31 ± 1.11	**0.010**
	2–6 Y	68	1.83 ± 1.17	1.10 ± 0.77	**0.000**
	6–18 Y	36	1.66 ± 0.80	1.01 ± 0.49	**0.000**
	0–18 Y	136	2.05 ± 1.55	1.17 ± 0.84	**0.000**
CCT (μm)	≤6 M	10	549.90 ± 47.15	533.00 ± 39.31	0.158
	6 M to 2 Y	22	572.09 ± 54.01	540.41 ± 49.23	**0.006**
	2–6 Y	67	565.06 ± 53.20	542.09 ± 47.60	**0.004**
	6–18 Y	36	565.22 ± 55.37	538.89 ± 38.37	**0.000**
	0–18 Y	135	565.13 ± 53.17	540.29 ± 44.61	**0.000**
ACD (mm)	≤6 M	10	2.65 ± 0.51	2.51 ± 0.26	0.325
	6 M to 2 Y	22	2.68 ± 0.64	2.69 ± 0.32	0.955
	2–6 Y	68	3.06 ± 0.62	2.86 ± 0.34	**0.009**
	6–18 Y	36	3.15 ± 0.58	3.02 ± 0.24	0.191
	0–18 Y	136	2.99 ± 0.63	2.85 ± 0.33	**0.006**

The data are presented as the means ± standard deviation (SD). Bold data are significant at P < 0.05. M: months; Y: years; D: diopters; Km: mean keratometry; Astig: anterior corneal astigmatism; CCT: central corneal thickness; ACD: anterior chamber depth.
